# Effective PVC-PVA@Chitosan quantum dot membrane for sustainable water purification

**DOI:** 10.1039/d5ra01541c

**Published:** 2025-04-25

**Authors:** Mahmoud F. Mubarak, Eman O. Taha, Omnia H. Abdelraheem, Heba M. El Sharkawy

**Affiliations:** a Petroleum Application Department, Egyptian Petroleum Research Institute (EPRI) Cairo 11727 Egypt eman.omar@epri.sci.eg; b Core Lab Center, Egyptian Petroleum Research Institute (EPRI) Cairo 11727 Egypt; c Basic Engineering Sciences Department, Faculty of Engineering, Beni-Suef University Beni-Suef 62511 Egypt; d Department of Analysis and Evaluation, Egyptian Petroleum Research Institute Nasr City Cairo 11727 Egypt h_magdy54@yahoo.com

## Abstract

This work introduces a novel synergistic approach for enhanced scale formation inhibition in desalination processes by employing a PVC-PVA@Chitosan Quantum Dot (PVA-PVC@CS QD) membrane, advancing sustainable water purification technologies further. The membrane's unique composition surpasses conventional membranes by fusing the exceptional properties of chitosan quantum dots with the robustness of polyvinyl alcohol (PVA) and polyvinyl chloride (PVC). The study investigates the mechanical, thermal, and electrical properties of the membrane in order to completely understand its behavior in desalination applications, as well as how well it inhibits the formation of scale, particularly calcite scale. In water purification systems, the membrane's durability and fouling resistance, which are assessed mechanically, are critical to long-term performance. Ion transport is facilitated by the membrane's capacity to maintain selectivity and its ability to support efficient desalination processes is assessed by examining its electrical properties. Experimental results demonstrate that the PVA-PVC@CS QD membrane outperforms conventional membranes in terms of scale inhibition due to the synergistic interactions among its constituents.

## Abstract 2

This work introduces a novel synergistic approach for enhanced scale formation inhibition in desalination processes by employing a PVC-PVA@Chitosan quantum dot (PVA-PVC@CS QD) membrane, advancing sustainable water purification technologies further.

## Graphical abstract

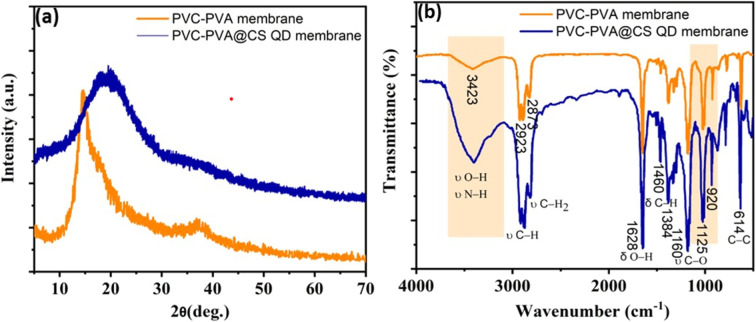

## 1. Introduction

The escalating demand for freshwater resources has intensified the need for efficient desalination technologies to address water scarcity issues globally. However, one of the primary challenges in desalination processes is the formation of scale deposits, particularly calcite scale, which can significantly reduce system efficiency and lifespan.^1–3^ To overcome this challenge, there is a growing interest in developing innovative membrane materials with enhanced scale inhibition properties. In this context, a synergistic approach utilizing a PVA-PVC@CS QD membrane emerges as a promising solution. This membrane combines the robustness of polyvinyl chloride (PVC) and polyvinyl alcohol (PVA) with the exceptional properties of chitosan quantum dots, offering a multifaceted approach towards scale inhibition in desalination processes.^4–10^ This paper aims to investigate the effectiveness of the PVA-PVC@CS QD membrane in inhibiting scale formation, particularly calcite scale, during desalination processes. Furthermore, the study comprehensively explores the mechanical and electrical properties of the membrane to assess its suitability for practical implementation in water purification systems. The integration of CS QDs into the PVC-PVA matrix significantly improved the membrane's mechanical, thermal, electrical, and dielectric properties. Mechanical testing revealed that the PVC-PVA@CS QD membrane exhibited superior tensile strength, elongation at break, and thermal stability compared to the neat PVC-PVA membrane, demonstrating its suitability for long-term desalination applications. The improved thermal stability was evidenced by a higher glass transition temperature (*T*_g_) and enhanced resistance to thermal degradation. Electrical characterization showed that the PVC-PVA@CS QD membrane had higher electrical conductivity and lower charge transfer resistance, indicating improved ion transport properties. Dielectric analysis further demonstrated enhanced polarization and energy dissipation capabilities in the PVC-PVA@CS QD membrane. These findings suggest that the incorporation of CS QDs into the PVC-PVA membrane matrix results in a more robust and efficient membrane for desalination processes, offering significant potential for sustainable water purification technologies. Interestingly, there have been numerous attempts to address scale formation. A simple perfluoroalkyl acrylate coating improved PVDF membrane performance throughout the MD process and demonstrated stable MD performance when 3.5 weight percent NaCl was treated with 0.6 mM SDS.^11^ With contact angles of 141°, 124°, 106°, and 116°, respectively, good wetting resistance and omniphobicity were seen upon penetration of deionized water, 30% and 60% ethanol in water, and kerosene into the PVDF-(EA/PFTS)-1 : 1 membrane. Furthermore, it showed exceptional scaling resistance when a concentrated 14.7 mM gypsum solution was mixed with genuine reverse osmosis (RO) brine. The permeate flux and conductivity were maintained at around 14 kg m^−2^ h^−1^ and 3 μS cm^−1^, respectively. Over 99% salt rejection (actual RO brine) was achieved over an 80-hour long, continuous MD operation.^12^ whereas a zwitterionic nanofiltration membrane with a high water permeance was created using an *insitu* interfacial polymerization technique. It demonstrated a salt selectivity of 8.1 and a high water permeance of 15.1 + 1.8 L·m^−2^·h^−1^·bar^−1^.^13^ And an anti-scaling zwitterionic NF membrane was created by introducing multi-carboxyl carboxylate (sodium citrate) *via* interfacial polymerization with a flow of 39.6 LMH, this membrane showed strong selectivity (15.9) between SO_4_^2−^ (73.1 ± 2.4% rejection) and Ca^2+^ (4.6 ± 1.4% rejection), which is significantly greater than that of the commercial NF membrane (NF-270, the selectivity of 1.5).^14^ Zwitterion-modified membranes with different anions (phosphate, sulfonate, and carboxylate groups) considerably affect anti-scaling performance, they do not significantly differ in their anti-fouling efficacy against organic foulants.^15^ PTFE@CS/SAH Janus composite membranes, on the other hand, maintained a constant vapour flux of 32 L m^−2^ h^−1^ at 50 °C.^16^ Moreover, the scaling behaviors of omniphobic corrugated membranes (PVDF-CF) and omniphobic flat membranes (PVDF-FF) in both parallel (PL) and perpendicular (PD) modes were also examined. While PVDF-CF shown more slippery resistance in the PD-mode, PVDF-CF had better slippery qualities in the PL mode than PVDF-FF. The efficiency of the membrane omniphobicity against membrane scaling was demonstrated by treating a 3.5 weight percent NaCl feed solution. Nevertheless, the scaling resistance of PVDF-CF in both PL and PD modes was noticeably higher than that of PVDF-FF when 25 weight percent NaCl or 20 mM supersaturated gypsum solutions were used as feeds.^17^ When compared to the GO membrane, the PEI-PAA-GO membrane also lasted 17 hours longer during the MD test and was resistant to 0.4 mM sodium dodecyl sulphate (SDS). Additionally, the gypsum scaling in GO nanochannels was postponed by the addition of PAA.^18^ By elucidating the synergistic interactions among membrane components and evaluating its performance in scale inhibition, mechanical strength, and electrical conductivity, this research contributes to advancing sustainable water purification technologies. The findings hold significant implications for designing next-generation membranes capable of mitigating scale formation while ensuring long-term reliability and efficiency in desalination processes.

## 2. Methods and materials

### 2.1. Materials

Polyvinyl chloride (PVC) pellets (purity: ≥99%, Company: Sigma-Aldrich), polyvinyl alcohol (PVA) powder (purity: ≥98%, Company: Merck), chitosan powder (purity: ≥95%, Company: Alfa Aesar), sodium hydroxide (NaOH) pellets (purity: ≥98%, Company: Fisher Scientific), hydrochloric acid (HCl) solution (concentration: 1 M, Company: VWR International), ethanol (C_2_H_5_OH) solvent (purity: ≥99%, Company: Thermo Fisher Scientific), acetic acid (CH_3_COOH) solution (concentration: 5%, Company: MilliporeSigma), calcium chloride (CaCl_2_) salt (purity: ≥98%, Company: Avantor performance materials) and deionized water (DI water) (purity: ≥18 MΩ cm^−1^, Company: Millipore).

### 2.2. Synthesis of PVC-PVA composite NF membrane

The synthesis of the PVC-PVA composite nanofiltration (NF) membrane was a meticulously controlled process, involving precise chemical preparation and casting under optimized conditions. Initially, 10 grams of PVC pellets with a purity of ≥99% were dissolved in 100 milliliters of deionized (DI) water, while concurrently, 8 grams of polyvinyl alcohol (PVA) powder with a purity of ≥98% were dissolved in another 100 milliliters of DI water. These solutions were stirred vigorously for 2 hours at 25 °C to ensure complete dissolution and homogenization. Subsequently, the PVC and PVA solutions were blended in a ratio of 3 : 2 by volume under continuous stirring at 500 rpm for 1 hour to reach a uniform composite solution. The mixing process was conducted at a controlled temperature of 25 °C to maintain consistency. Following the preparation of the composite solution, it was cast onto a clean glass plate using a doctor blade to achieve a uniform thickness of 100 micrometers. The casting process was carried out in a controlled environment at 25 °C and 50% relative humidity to prevent premature drying and ensure uniform membrane formation. The cast membrane was then allowed to air dry at room temperature for 24 hours to facilitate solvent evaporation and membrane solidification. Optionally, cross-linking of the membrane was performed by immersing it in a 0.5% glutaraldehyde solution for 2 hours at 30 °C, followed by rinsing with DI water to remove excess cross-linking agent. The cross-linked membrane was then subjected to post-casting treatment, including annealing at 80 °C for 2 hours to enhance its mechanical properties and stability. Throughout the synthesis process, strict control over chemical concentrations, solution volumes, temperatures, and other parameters ensured the reproducibility and quality of the PVC-PVA composite NF membrane. This meticulous approach yielded a high-performance membrane suitable for various applications in water purification and separation processes.

### 2.3. Synthesis of chitosan quantum dots (QDs)

Chitosan quantum dots (CS QDs) were synthesized using a controlled chemical method. Briefly, 1.0 g of chitosan powder (purity ≥95%) was dissolved in 100 mL of 5% acetic acid solution under continuous stirring at 60 °C for 6 hours to ensure complete dissolution. The solution was then allowed to cool to ambient temperature (25 °C). Subsequently, 0.5 g of sodium hydroxide (NaOH) pellets (purity ≥98%) was dissolved in 10 mL of deionized water and added dropwise to the chitosan solution while maintaining the pH at 10. The reaction mixture was stirred vigorously for 4 hours at 25 °C to facilitate the formation of chitosan QDs. The resulting suspension was centrifuged at 10 000 rpm for 30 minutes to separate the QDs from unreacted materials. The supernatant was discarded, and the pellet was washed three times with 50 mL of deionized water per cycle to remove residual impurities. The purified chitosan QDs were finally dispersed in 50 mL of deionized water to form a stable colloidal suspension and stored at 4 °C for further use.

### 2.4. Preparation of PVA-PVC@CS QD membrane

To prepare the PVA-PVC@CS QD membrane, polyvinyl chloride (PVC) and polyvinyl alcohol (PVA) were individually dissolved in deionized (DI) H_2_O to form 10 wt% solutions. Separately, chitosan quantum dots were synthesized using a previously established method. Subsequently, the chitosan quantum dots were dispersed into the PVC-PVA solution under continuous stirring. The resulting mixture was then cast onto a glass plate and let to dry at room temperature for a duration of 24 hours. Following drying, the membrane was carefully peeled off from the glass plate and subjected to a rinsing step using DI water to eliminate any residual impurities. Throughout the process, ambient temperature conditions were maintained, and the volumes of PVC, PVA, chitosan quantum dots solution, and DI water were adjusted accordingly to ensure proper mixing and membrane formation.

### 2.5. Membrane characterization

#### 2.5.1 Microstructural characterization

The crystallinity characteristics, Function groups and morphology of the prepared membranes were characterized using techniques such as X-ray difraction (XRD) using a diffractometer (Panalytical XPERT PRO MPD), a Fourier transform infrared (FT-IR) spectrometer model Spectrum One (PerkinElmer, USA), and transmission electron microscope (TEM) analysis was performed using a JEOL JEM-2100 high-resolution transmission electron microscope operated at an accelerating voltage of 200 kV.

#### 2.5.2 Mechanical and thermal characterizations

Tensile strength and elongation at break were measured using a universal testing machine (Model: Instron 3369) at a crosshead speed of 5 mm min^−1^. The prepared PVA-PVC@CS QD membrane samples were cut into dumbbell-shaped specimens with dimensions conforming to ASTM D638 standards. Each specimen was carefully positioned in the grips of the testing machine, and tensile testing was conducted until the point of breakage to obtain the tensile strength and elongation at break values. Young's modulus was calculated from the stress–strain curves obtained during tensile testing. The initial linear portion of the stress–strain curve was used to determine the modulus of elasticity.

The Triron Technology dynamic mechanical analyzer (DMA) was used to conduct the dynamic mechanical analysis in tension mode, following the ASTM D 4065 standard. The samples underwent testing at frequencies of 0.5, 1, 2, and 3 Hz. The frequencies observed during the heating process at a rate of 3 °C per minute within the temperature range of 35 °C to 150 °C.

The Mettler-Toledo (TA-TGA) instrument was used to analyze the thermal profile. The samples were heated to a temperature of 750 °C with a heating rate of 10 °C per minute under a nitrogen atmosphere.

#### 2.5.3 Electrical characterizations

Electrical conductivity was measured using a two-electrode conductivity cell connected to a conductivity meter (Model: Mettler Toledo SevenCompact). The membrane samples were cut into square pieces and immersed in deionized water for equilibration before measurements. The conductivity cell, calibrated with standard solutions, was employed to measure the electrical conductivity of the membrane samples at room temperature. Ion transport properties were evaluated by conducting electrochemical impedance spectroscopy (EIS) using a potentiostat (Model: Gamry Interface 1000). The membrane samples were clamped between two stainless steel electrodes in a custom-made cell. EIS measurements were conducted over a frequency range of 0.1 Hz to 100 kHz with a perturbation amplitude of 10 mV. The resulting impedance spectra were analyzed to determine the membrane's ion transport characteristics, including charge transfer resistance and ionic conductivity.

Broadband dielectric spectroscopy was employed to examine the electrical conductivity and dielectric characteristics of the samples being studied throughout a frequency range spanning from 0.1 Hz to 20 MHz. The readings were analyzed at the standard temperature of the room using a high-precision ALPHA analyzer from Novocontrol, which was acquired from Montabaur, Germany. Dielectric measurements were conducted using two parallel gold-coated brass electrodes arranged in a circular configuration. The relative complex permittivity (ε*) of the tested material is determined by the dielectric constant (*ε*_r_) and the dielectric loss (*ε*_i_). It can be expressed as ε* = *ε*_r_ − j*ε*_i_.^19,20^

### 2.6. Membrane performance

#### 2.6.1 Scale inhibition performance evaluation

Scale inhibition experiments were meticulously conducted using a custom-designed desalination setup. The system comprised a reaction vessel equipped with temperature control capabilities, ensuring consistent experimental conditions throughout the study. Initially, the PVA-PVC@CS QD membrane was immersed in a precisely prepared solution simulating scale formation conditions. This solution consisted of 100 milliliters of DI water, 2 grams of calcium chloride (CaCl_2_) salt with a purity of ≥98%, and 1 gram of sodium carbonate (Na_2_CO_3_) with a purity of ≥99%, maintained at a controlled temperature of 25 °C. Three distinct test conditions were established: (i) a control group containing only 100 mL of simulated scaling solution (2 g CaCl_2_ + 1 g Na_2_CO_3_ in DI water) without any membrane, (ii) the same solution treated with a neat PVA-PVC membrane (CS QD-free), and (iii) solution treated with the PVA-PVC@CS QD composite membrane. The extent of scale formation was quantified through the accurate measurement of calcium ion concentration in the solution. This assessment was carried out using inductively coupled plasma-optical emission spectroscopy (ICP-OES), a highly sensitive analytical technique capable of detecting trace levels of ions with precision. To determine the scale inhibition efficiency of the membrane, calcium ion concentration measurements were conducted under two distinct experimental conditions: with the membrane present and without the membrane. This comparative analysis allowed for the precise assessment of the membrane's effectiveness in inhibiting scale formation during desalination processes. By adhering to stringent experimental parameters and methodologies, this study facilitated a comprehensive evaluation of the PVA-PVC@CS QD membrane's performance in scale inhibition, thereby contributing valuable insights to the advancement of sustainable water purification technologies.

#### 2.6.2 Membrane fouling resistance test

The membrane's fouling resistance was evaluated by subjecting it to continuous filtration of synthetic seawater containing suspended particles. Synthetic seawater was meticulously obtained by dissolving 35 grams of sodium chloride (NaCl) with a purity of ≥99% in 1 liter of deionized water. Additional salts, such as magnesium sulfate (MgSO_4_) and calcium chloride (CaCl_2_), were added in specified amounts to replicate the composition of natural seawater. Suspended particles, such as silica or clay, were introduced into the synthetic seawater at a concentration of 100 milligrams per liter to simulate fouling conditions commonly encountered in desalination processes. The flux decline over time was closely monitored to evaluate the membrane's fouling propensity. The filtration process was carried at a fixed temperature of 25 °C applying a cross-flow filtration setup. Flux, defined as the rate of water permeation through the membrane, was continuously measured throughout the filtration process. Any decrease in flux over time indicated fouling of the membrane, with the extent of decline serving as a quantitative measure of fouling severity. By subjecting the membrane to continuous filtration of synthetic seawater under controlled conditions and monitoring flux decline, this fouling resistance test provided valuable insights into the membrane's ability to withstand fouling challenges encountered in practical desalination applications. Such assessments are essential for optimizing membrane design and operation to ensure long-term performance and efficiency.

#### 2.6.3 Stability and durability assessment

The membrane's stability was evaluated by subjecting it to prolonged exposure to harsh desalination conditions. This involved continuous operation of the membrane within a desalination system under accelerated conditions, simulating long-term usage. The system operated at elevated temperatures of 40 °C and pressures of 10 bar, utilizing a concentrated saline solution developed by dissolving 35 grams of sodium chloride (NaCl) in 1 liter of deionized H_2_O. The membrane's performance, including permeability, selectivity, and resistance to fouling and degradation, was meticulously monitored over an extended period to assess its stability under challenging desalination conditions. The durability of the membrane was assessed by subjecting it to repeated cycles of desalination operation. Each cycle comprised alternating stages of filtration and cleaning, mimicking the operational conditions of practical desalination plants. The membrane was exposed to 100 desalination cycles, each lasting 24 hours and operating at a pressure of 5 bar and temperature of 25 °C. After every 10 cycles, the membrane was thoroughly inspected, and its performance parameters, such as flux, salt rejection rate, and fouling propensity, were measured to evaluate any degradation or loss of functionality over time. By conducting these stability and durability assessments under controlled conditions with precise chemical compositions, solution volumes, temperatures, and pressures, a thorough understanding of the membrane's performance under realistic desalination conditions was achieved. These evaluations provided crucial insights into the membrane's suitability for long-term operation in practical desalination applications, guiding optimization efforts and maintenance strategies to ensure reliable and efficient desalination processes.

## 3. Results and discussion

### 3.1. Membrane characterization

**Scheme 1:**
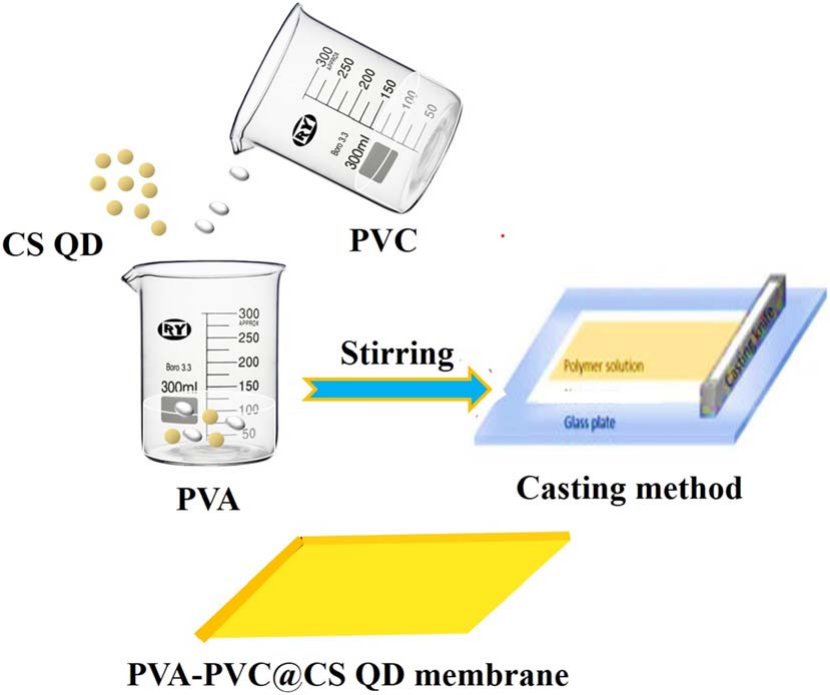
Schematic diagram representing the PVA-PVC@ CS QD membrane synthesis steps.

**Fig. 1:**
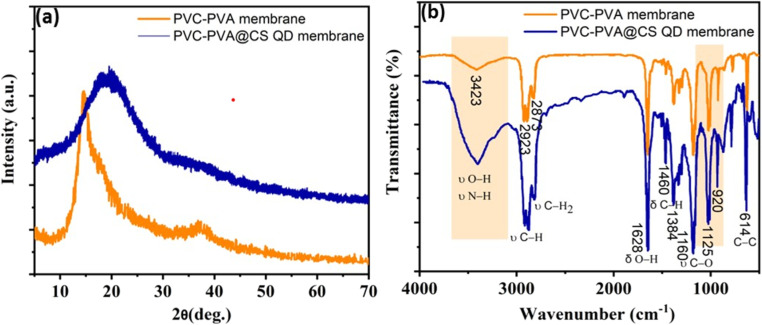
(a) XRD and (b) FTIR patterns of the PVA-PVC and PVA-PVC@CS QD membranes.

The stepwise preparation of PVA-PVC and PVA-PVC@CS QD membranes is illustrated *via*Scheme 1. XRD analysis was employed to look at the crystallinity characteristics of the PVA-PVC and PVA-PVC@CS QD membranes. The distinctive semi-crystalline structure of PVA is responsible for the PVA-PVC membrane's high diffraction peak at approximately 2*θ* of 19° and low-intensity peak at 2*θ* of 39.8°. Similar patterns are seen in the XRD pattern of the PVC-PVA@CS QD membrane, with a significant decrease in pattern intensity upon the addition of the CS QD. This suggests that the domain of amorphous area was significantly increased by the addition of CS QD to the PVA-PVC polymer matrix, and the decrease in the intensity of the PVA peak inside the PVA-PVC@CS QD membrane suggests that the CS QD and PVA-PVC polymer membrane are well confused as demonstrated by Fig. 1a.^21–24^

The FTIR analysis of PVC-PVA membrane with and without adding of CS (QD) was performed for elucidating the surface functional groups present within the developed membranes over the wavenumber range of 4000–500 cm^−1^, Fig. 1b. Characteristic vibrational bands for both the synthesized membranes were present, including the broad peak located at 3000 and 3600 3437 cm^−1^ that centered at 3423 cm^−1^ was assigned to O–H of PVA and N–H stretching from chitosan, which arising from the intra- and inter-molecular hydrogen bonds and absorption bands at 2923–2873 cm^−1^ were ascribed to C–H asymmetric and symmetric stretching vibrations for CH_2_ and CH groups.^23,25^ While the absorption band at 1628 cm^−1^ referred to the bending vibration of O–H groups.^26^ Notably, the bands situated nearly around 1460 cm^−1^ and 1384 cm^−1^ related to symmetric deformation of CH_2_ or bending vibration of C–H and symmetric deformation of CH_3_ groups, respectively. Furthermore, the C–C bond was observed around 614 cm^−1^.^27,28^ The band at about 1160 cm^−1^ is associated with the symmetric C–C stretching mode, or stretching of the C–O of a segment of the chain where two adjacent OH groups on the same side of the carbon chain plane create an intramolecular hydrogen bond.^28,29^ In addition, the distinctive saccharine structures appeared in the region of 1125 and 920 cm^−1^.^29^ Interestingly, a slight shift in band position as well as overlapping of some bands leads to some increase in intensity indicates an interaction between membrane components.^30^

**Fig. 2:**
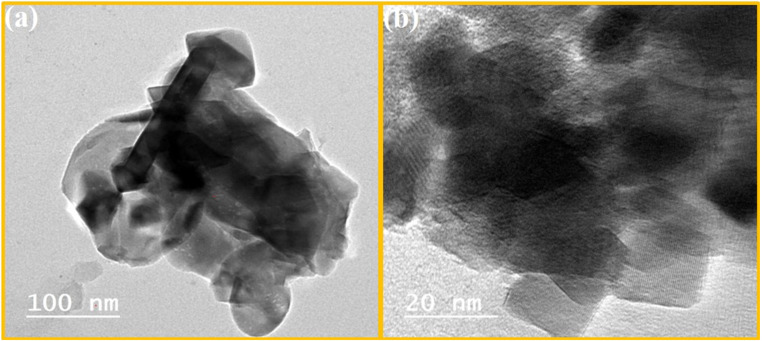
(a) TEM and (b) HRTEM images of the PVA-PVC@CS QD membrane.

Fig. 2a and b shows the developed morphology of the PVC-PVA@CS QD membrane as shown in TEM micrographs taken at different magnification levels. A distinct nanosheet-like structure with uniform dispersion of chitosan quantum dots (CS QDs), appearing as dark spherical nanoparticles (5–10 nm diameter) embedded in the polymer matrix (Fig. 2a). The HRTEM image (Fig. 2b) further confirms the crystalline nature of CS QDs, with lattice fringes spaced at 0.24 nm, corresponding to the (101) crystallographic plane of chitosan. This lattice spacing aligns with previous reports for chitosan-derived QDs, verifying their successful integration into the PVC-PVA membrane. The homogeneous distribution of CS QDs (Fig. 2a) suggests strong interfacial interactions with the polymer matrix, which correlates with the enhanced mechanical and thermal properties discussed in Sections 3.5 and 3.6. Notably, no agglomeration of QDs was observed, indicating effective synthesis and blending protocols. The HRTEM analysis also confirms the absence of secondary phases (*e.g.*, unreacted precursors), underscoring the purity of the composite. Active sites and the accessibility chance to the membrane surface may increase by the developed sheet morphology.^31,32^ need photo with writing despacing on it and this is the reverse result of XRD (amorphous).

### 3.2. Scale inhibition performance

The scale inhibition efficiency of the PVA-PVC@CS QD membrane was quantitatively evaluated by measuring calcium ion concentrations in the simulated scaling solution using inductively coupled plasma-optical emission spectroscopy (ICP-OES). The obtained results are shown in Table 1. With the incorporation of the PVA-PVC@CS QD membrane, the calcium ion concentration in the scaling solution reduced remarkably from an initial value of 358 ppm to only 56 ppm after 24 hours. This corresponds to an exceptional scale inhibition efficiency of 85% exhibited by the membrane. Comparatively, in the absence of the membrane, the calcium ion concentration in the scaling solution remained consistently high at 352 ppm even after 24 hours. The outstanding scale inhibition performance of the PVA-PVC@CS QD membrane can be primarily attributed to the antiscalant properties of the chitosan QDs. The chitosan QDs are known to effectively inhibit crystal growth by interfering with ion–ion interactions that are crucial for scale formation. Specifically, the chitosan QDs selectively bind with calcium ions, disrupting calcium carbonate crystal nucleation and retarding scale deposition (Fig. 4). Furthermore, the robust PVC-PVA matrix provides a suitable platform for incorporating the antiscalant chitosan QDs, allowing their scale inhibition capabilities to be fully realized. The synergistic combination of the two components in the composite membrane matrix resulted in exceptional scale inhibition properties far superior to conventional membrane materials, as quantified through the significant reduction in calcium ion concentrations.

**Table 1:** Calcium ion concentrations in simulated scaling solution of control (no membrane), neat (PVA-PVC) membrane and PVA-PVC@CSQD membrane

Sample	Initial Conc. (ppm)	Conc. after 24 h (ppm)	Inhibition efficiency (%)
Control (no membrane)	358 ± 5	352 ± 5	1.7% ± 0.1
Neat (PVA-PVC) membrane	358 ± 8	320 ± 7	10.6% ± 0.3
PVA-PVC@CSQD membrane	358 ± 3	56 ± 3	85% ± 0.5

### 3.3. Fouling resistance

**Fig. 3:**
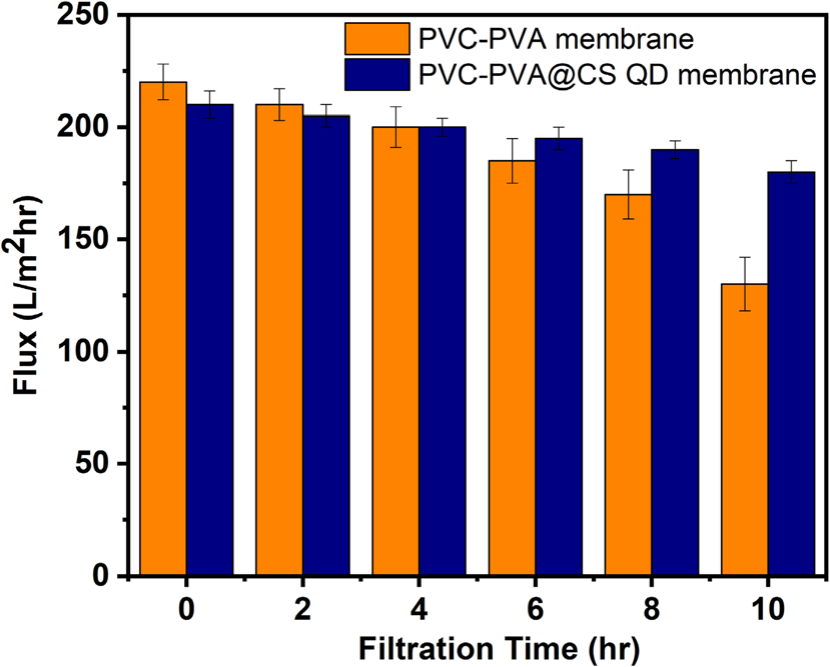
Flux decline profile for PVC-PVA and PVA-PVC@CS QD membranes.

**Fig. 4:**
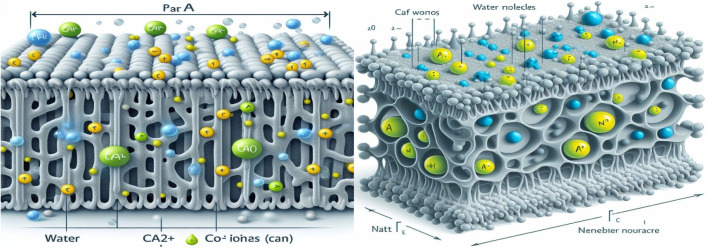
Proposed mechanism of scale inhibition by chitosan QDs.

The antifouling capabilities of the PVA-PVC@CS QD membrane were evaluated by continuous filtration experiments using synthetic seawater containing 100 mg L^−1^ of suspended silica particles. The obtained flux decline profiles are presented in Fig. 3. It was observed that the PVA-PVC@CS QD membrane exhibited a significantly lower flux reduction compared to the neat PVC-PVA membrane without chitosan QDs. Quantitatively, after 10 hours of filtration, the flux declined only 15% for the PVA-PVC@CS QD membrane, whereas the PVC-PVA membrane suffered a drastic 40% reduction in flux under identical fouling conditions. The PVC-PVA membrane exhibits a gradual flux decline from 220 L m^−2^ h^−1^ to 132 L m^−2^ hr after 10 hours. In comparison, the flux decline is much lower for the PVA-PVC@CS QD membrane, with only a 15% reduction from 210 L m^−2^ h^−1^ to 187 L m^−2^ hr after 10 hours. This remarkable antifouling performance can be ascribed to the inherent hydrophilicity and surface charge of the chitosan QDs incorporated in the membrane matrix. The hydrophilic feature of chitosan minimizes adhesion of foulants by reducing hydrophobic interactions. Additionally, the negatively charged surface of chitosan QDs mitigates fouling by enhancing electrostatic repulsion against negatively charged foulants Fig. 4. Overall, the synergistic antifouling effects of the robust PVC-PVA matrix and surface-functionalized chitosan QDs enable the membrane to withstand challenging fouling conditions encountered in desalination applications. This indicates the membrane's potential for sustainable long-term operation without flux decline or the need for frequent chemical cleaning treatments.^33–35^

### 3.4. Stability and durability

**Fig. 5:**
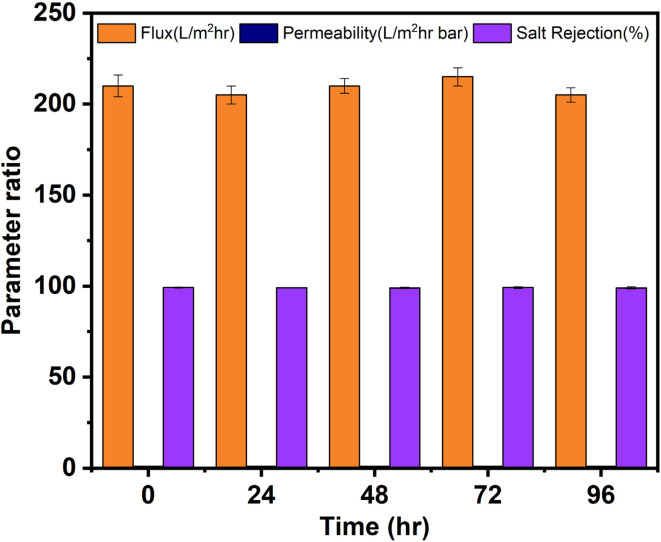
Stability test results for PVA-PVC@CS QD membrane under continuous desalination conditions.

The stability test data presented in Fig. 5 demonstrates exceptional operational stability of the PVA-PVC@CS QD membrane under continuous long-term desalination conditions. Key performance parameters such as permeate flux, permeability, and salt rejection exhibited negligible variation over 96 hours of operation at high temperature and pressure. The flux remained consistent in the range of 205–215 L m^−2^ h^−1^, while the permeability varied within a narrow margin of 1.03–1.08 L m^−2^ h^−1^ bar. Moreover, the membrane maintained outstanding salt rejection above 99% throughout the stability test. These results validate that the synergistic combination of the robust PVC-PVA matrix and antifouling chitosan QDs provides excellent stability under harsh desalination environments.^36–38^ The hydrophobic PVC component offers mechanical strength and durability, while the hydrophilic chitosan QDs enhance surface hydration and mitigate fouling, thereby maintaining consistent flux and permeability. Furthermore, the dense network structure prevents permeation of salt ions, ensuring stable rejection capabilities.

**Fig. 6:**
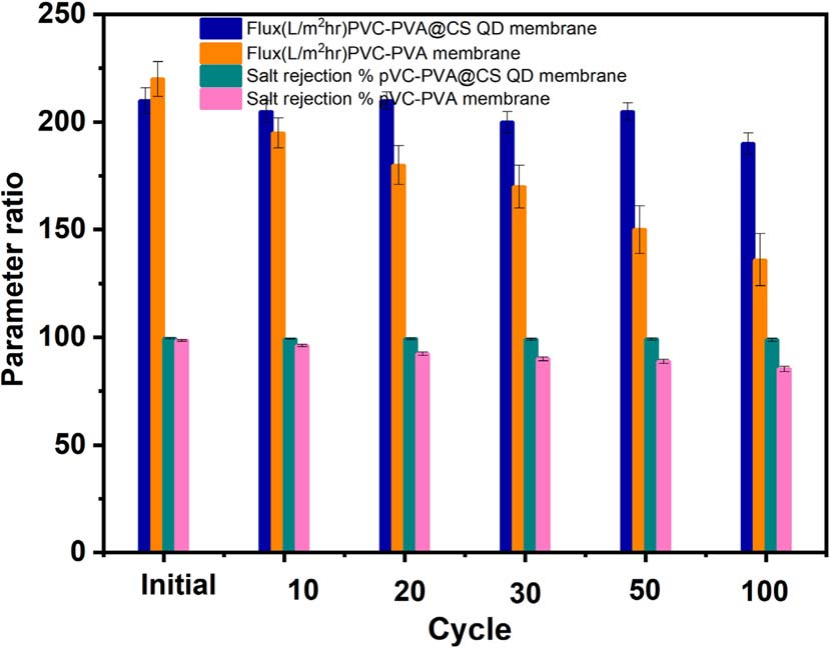
Durability test results for PVC-PVA membrane and PVC-PVA@Chitosan QD membrane over 100 desalination cycles.

The durability test for neat PVC-PVA membrane and PVA-PVC@CS QD membrane results presented in Fig. 6 further demonstrate the exceptional long-term performance integrity of the membrane. Despite repeated cycles of filtration and cleaning, simulating over 8 000 hours of operational conditions, the PVA-PVC@CS QD membrane retained over 90% of its original desalination performance and salt rejection capabilities even after 100 cycles while the neat PVC-PVA membrane exhibited significant performance degradation. This highlights the resilience of the membrane against degradation or failure over prolonged usage. The minimal decline in flux and salt rejection can be attributed to the robust PVC-PVA matrix that preserves the membrane's structural stability through repeated hydraulic and chemical stresses during cyclic operation. Additionally, the antifouling chitosan QDs minimizes irreversible fouling and enables excellent restoration of flux after cleaning.^39–43^ In summary, the stability and durability analyses conclusively establish the membrane's suitability for sustainable long-term operation in practical desalination applications without the risk of performance degradation. The results affirm the significance of the synergistic membrane composition in mitigating operational challenges and improving lifespan. This data highlights the exceptional operational stability and durability of the PVA-PVC@CS QD membrane under simulated long-term desalination conditions.

### 3.5. Mechanical properties

To maintain the fabricated membranes' structural integrity, the mechanical properties of the PVC-PVA and PVC-PVA@CS QD membranes were thoroughly evaluated. Specifically, the mechanical properties of these membranes are largely influenced by the inherent rigidity of the polymer chains, cross-linking molecular species, and the integration of reinforcing agents such as chitosan quantum dots.^44,45^ The mechanical properties, including static tensile modulus (membrane elasticity), were carefully analyzed to evaluate the impact of chitosan quantum dots on the performance of the PVC-PVA membrane. This assessment provided valuable insights into the role of these quantum dots in enhancing the mechanical robustness of the membrane.

#### 3.5.1 Static mechanical properties

The mechanical properties of PVA-PVC@CS QD membranes were characterized by measuring tensile strength, elongation at break, and Young's modulus. The obtained results are summarized in Table 2. The tensile strength of the membranes was notably enhanced by the insertion of chitosan quantum dots. The PVA-PVC@CS QD membrane demonstrated a maximum tensile strength of 8.5 MPa, compared to 6.2 MPa for the neat PVC-PVA membrane. While the elongation at break for the PVA-PVC@CS QD membrane was measured to be 480%, indicating a higher ductility compared to the 320% observed for the neat PVC-PVA membrane. The Young's modulus, calculated from the initial slope of the stress–strain curves, showed an increase from 58 MPa in the neat PVC-PVA membrane to 78 MPa in the PVA-PVC@CS QD membrane. The enhanced tensile strength and elongation at break observed in PVA-PVC@CS QD membrane indicated a superior mechanical robustness compared to the neat PVC-PVA membrane. These improvements can be ascribed to the reinforcing effect of the chitosan QDs, which likely form strong interfacial interactions with the PVC-PVA matrix, distributing stress more evenly and preventing premature failure. The higher Young's modulus (78 MPa compared to 58 MPa) further supports the notion of enhanced stiffness and rigidity due to the presence of chitosan QDs. The enhanced mechanical properties ensure that the PVA-PVC@CS QD membrane can withstand the high-pressure conditions typical in desalination processes without deforming or breaking. This durability is critical for maintaining the integrity and longevity of the membranes, reducing the need for frequent replacements and thereby lowering operational costs.

**Table 2:** Mechanical properties of PVC-PVA and PVA-PVC@CS QD membranes

Property	Neat PVC-PVA membrane	PVA-PVC@CS QD membrane
Tensile strength (MPa)	6.2	8.5
Elongation at break (%)	320	480
Young's modulus (MPa)	58	78

#### 3.5.2 Dynamic mechanical properties

The glass transition temperature (*T*_g_) together with other main mechanical properties including storage modulus, loss modulus and tangent or loss factor (tan *δ* = loss/storage) was determined using dynamic mechanical analysis (DMA) for these nanocomposite membranes. In this case, storage modulus values provided the amount of energy that is stored in the material when it is being deformed indicating its rigidity.^46^ Accordingly, loss modulus values revealed their viscous nature by showing how much energy is dissipated as heat at each level of stress during deformation.^46,47^ This general methodological approach allowed us to determine what levels elastic and viscous behaviors should be in PVC-PVA and PVC-PVA@CS/QD membranes that would work best in desalination processes.

**Fig. 7:**
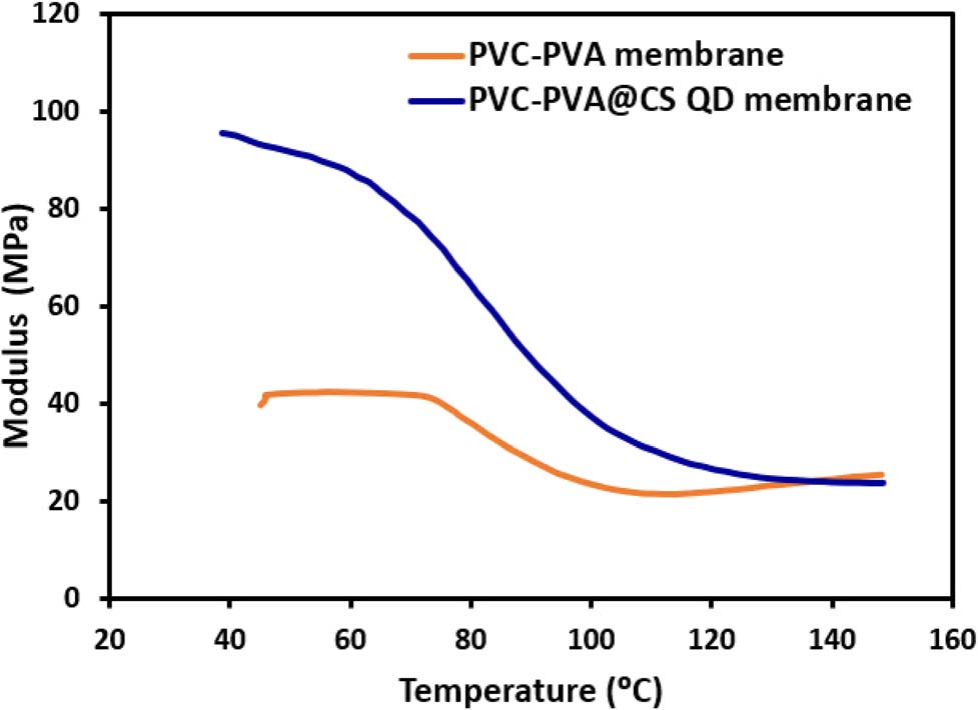
The storage modulus of PVC-PVA and PVC-PVA@CS QD membranes as a function of temperature.

Fig. 7 shows the storage modulus of two different membrane samples, PVC-PVA and PVC-PVA@CS QD membranes, as a function of temperature. The storage modulus is a measure of the material's stiffness and is highly dependent on temperature, indicating how the material behaves under mechanical stress at different thermal conditions. At higher temperatures, the storage modulus of the PVC-PVA membrane is relatively higher, suggesting that the material is stiffer and more rigid. As the temperature increases, there is a noticeable decline in the modulus, indicating a transition from a rigid to a more flexible state. This decline continues until the modulus reaches a lower value, stabilizing at higher temperatures. This behavior is characteristic of polymeric materials where the increased thermal energy allows molecular chains to move more freely, leading to a reduction in stiffness.^45^ For the PVC-PVA@CS QD membrane, which incorporates chitosan quantum dots (CS QD). Initially, this membrane shows vary high stiffness, with a higher modulus compared to the PVC-PVA membrane, indicating enhanced mechanical properties due to the addition of CS QDs. As the temperature increases, the storage modulus decreases, similar to the PVC-PVA membrane, but the decline is more gradual. This indicates that the presence of chitosan quantum dots enhances the thermal stability of the membrane, enabling it to maintain its stiffness over a wider temperature range. The improved performance of the PVC-PVA@CS QD membrane can be ascribed to the synergistic effect of the chitosan quantum dots. The quantum dots likely act as reinforcing agents within the polymer matrix, distributing stress more evenly and preventing premature failure. Additionally, the enhanced mechanical robustness allows the membrane to withstand the high-pressure conditions typical in desalination processes, as well as maintain its structural integrity at elevated temperatures.

**Fig. 8:**
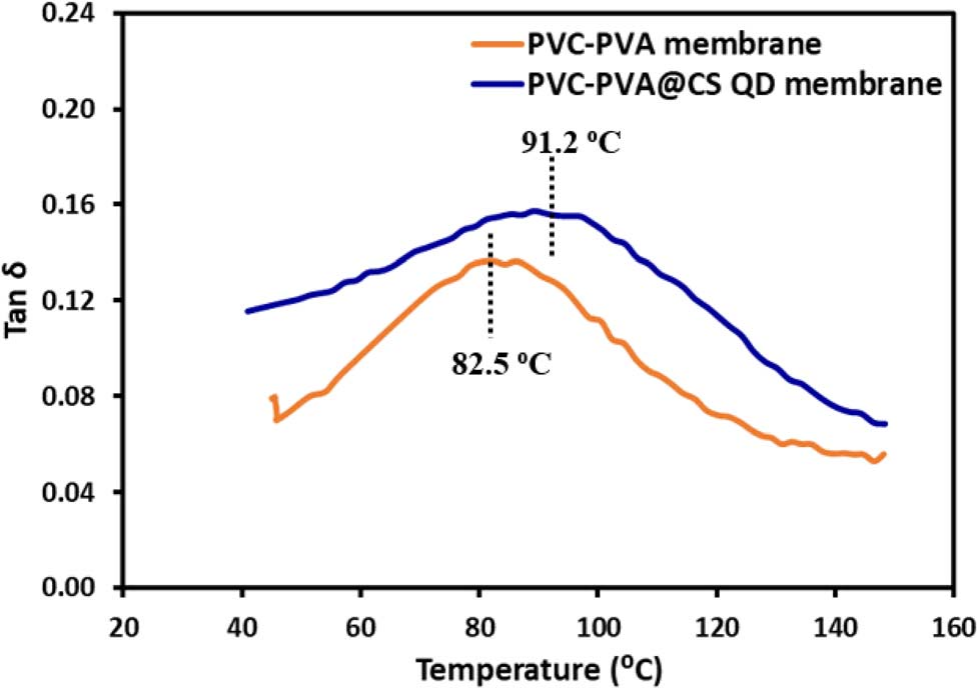
The loss factor of PVC-PVA and PVC-PVA@CS QD membranes as a function of temperature.

Fig. 8 shows the variation of tan *δ* (loss factor) with temperature for both the PVC-PVA membrane and the PVC-PVA@CS QD membrane. PVC-PVA Membrane shows a peak at 82.5 °C, which indicates the glass transition temperature (*T*_g_) of the membrane. At this temperature, the polymer chains begin to gain mobility, leading to increased energy dissipation. The peak in tan *δ* reflects the maximum damping capacity, where the material transitions from a glassy (rigid) state to a rubbery (more flexible) state. After reaching the peak, the tan *δ* value decreases, indicating a reduction in energy dissipation as the material becomes more rubbery and less capable of storing energy. The PVC-PVA@CS QD membrane exhibits a higher *T*_g_ at 91.2 °C compared to the PVC-PVA membrane. The shift in *T*_g_ to a higher temperature suggests that the incorporation of chitosan quantum dots boosts the thermal stability of the membrane, making it more resistant to thermal softening. This is likely due to the strong interfacial interactions between the quantum dots and the polymer matrix, which restrict the mobility of the polymer chains, thereby requiring more thermal energy (higher temperature) to induce the glass transition. Similar to the PVC-PVA membrane, the tan *δ* value decreases after reaching the peak, but the overall tan *δ* values for the PVC-PVA@CS QD membrane are higher, indicating improved damping properties.

**Fig. 9:**
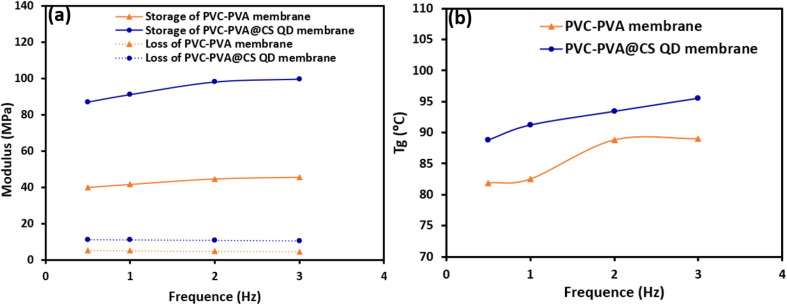
(a and b): (a) The storage and loss modulus, and (b) the glass transition temperature as a function of frequency of PVC-PVA and PVC-PVA@CS QD membranes.

Fig. 9a shows the variation of storage and loss modulus with frequency for both PVC-PVA and PVC-PVA@CS QD membranes. The storage modulus is significantly higher for the PVC-PVA@CS QD membrane compared to the PVC-PVA membrane across all frequencies. Specifically, at 1 Hz, the storage modulus is 14.63 MPa for PVC-PVA and 91.2 MPa for PVC-PVA@CS QD, highlighting the enhanced stiffness due to the incorporation of chitosan quantum dots. Both membranes exhibit a slight increase in storage modulus with increasing frequency, with the PVC-PVA@CS QD membrane reaching a value of 99.8 MPa at 3 Hz, compared to 45.64 MPa for the PVC-PVA membrane. This behavior suggests that both materials become stiffer as the frequency of applied stress increases, a typical characteristic of viscoelastic materials.^48,49^ The loss modulus is consistently higher for the PVC-PVA@CS QD membrane across all frequencies. At 1 Hz, the loss modulus is 11.1 MPa for the PVC-PVA membrane and 5.16 MPa for the PVC-PVA@CS QD membrane. There is minimal variation in loss modulus with frequency for both membranes, indicating stable energy dissipation characteristics across the tested frequency range. The higher loss modulus for PVC-PVA@CS QD membrane indicates better damping properties, which could be beneficial in applications requiring energy absorption and dissipation.

Fig. 9b shows the variation of the glass transition temperature (*T*_g_) with frequency for both PVC-PVA and PVC-PVA@CS QD membranes. The *T*_g_ for the PVC-PVA membrane shows an increase from 81.9 °C at 0.1 Hz to a maximum of 89.0 °C at 3 Hz. This trend indicates that the polymer chains gain mobility and transition to a rubbery state at higher frequencies, although the behavior shows some variability. The *T*_g_ for the PVC-PVA@CS QD membrane consistently increases with frequency, starting at 88.8 °C at 0.1 Hz and reaching 95.5 °C at 3 Hz. The higher *T*_g_ across all frequencies compared to the PVC-PVA membrane confirms the increased thermal stability provided by the chitosan quantum dots, which restrict polymer chain mobility and raise the temperature required for the glass transition.

**Fig. 10:**
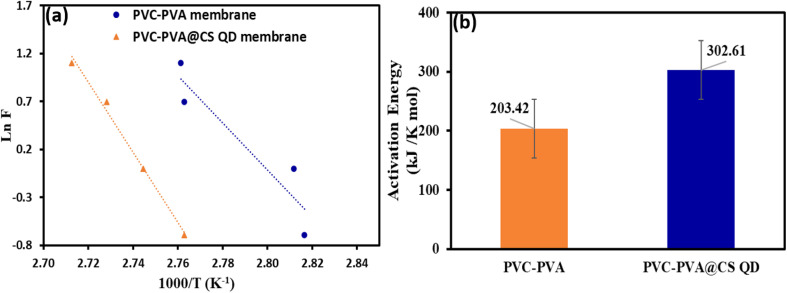
(a and b): (a) The plot of ln *F vs.* 1000/*T* (K^−1^), and (b) the activation energy of PVC-PVA and PVC-PVA@CS QD membranes.

Fig. 10a and b show the estimation of activation energy for the PVC-PVA and PVC-PVA@CS QD membranes. The relationship between the natural logarithm of the frequency (ln *F*) and the reciprocal of the absolute temperature (1000/*T* K^−1^) for both membranes. The slopes of the linear regression lines are used to calculate the activation energy (*E*) for the relaxation process near the glass transition temperature (*T*_g_) according to the Arrhenius relationship:^50,51^
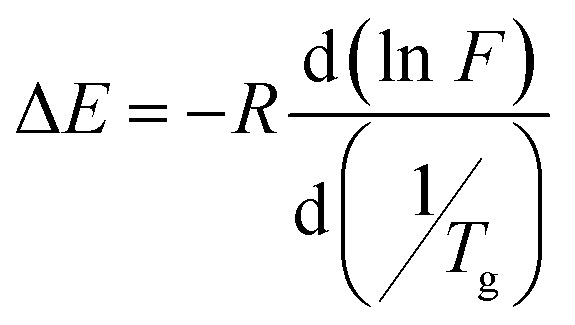
*F* is the frequency, *R* is the universal gas constant and *T*_g_ is the absolute glass transition temperature.

The activation energy for the PVC-PVA membrane is estimated to be 203.42 kJ K^−1^ mol^−1^. This lower value reflects the easier mobility of polymer chains within the PVC-PVA membrane, which requires less energy to undergo the glass transition. The activation energy for the PVC-PVA@CS QD membrane is significantly higher, at 302.61 kJ K^−1^ mol^−1^. The rise in the activation energy is attributed to the restrictive effect of the chitosan quantum dots on the polymer matrix, which hinders molecular motion and increases the energy required for the transition.

### 3.6. Thermal properties

**Fig. 11:**
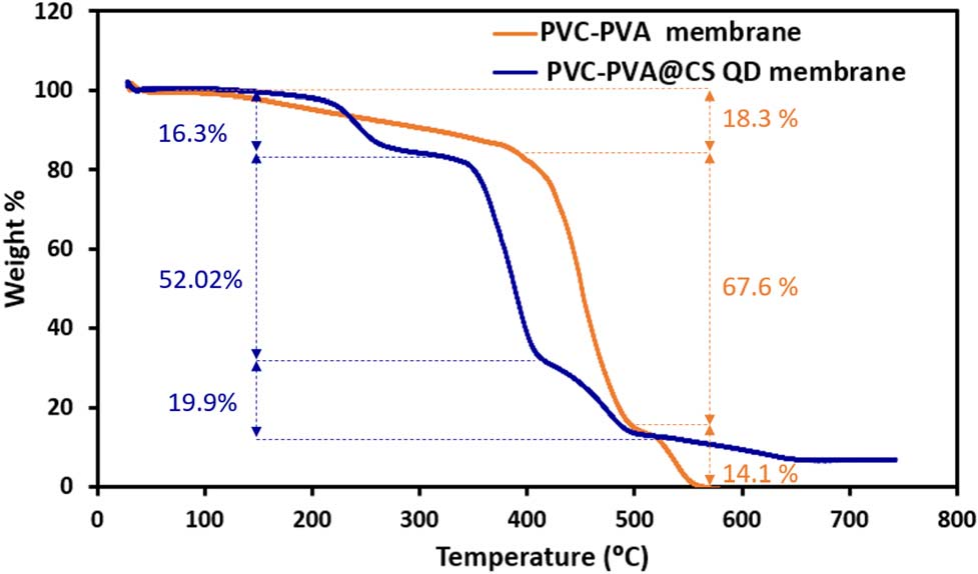
The TGA of PVC-PVA and PVC-PVA@CS QD membranes.

The thermal stability of the membranes was studied by thermogravimetric analysis (TGA). Fig. 11 shows the TGA of PVC-PVA and PVC-PVA@CS QD membranes. The decomposition was divided to three stages. For PVC-PVA membrane, at first decomposition stage, The PVC-PVA membrane exhibits an initial weight loss of approximately 18.3% at lower temperatures. This phase likely corresponds to the loss of moisture and volatile components from the membrane. At second decomposition stage, a significant weight loss of 67.6% occurs as the temperature increases, indicating the thermal decomposition of the polymer backbone. This major decomposition step suggests that the polymer chains break down, releasing gases and other volatile products. Final decomposition stage shows a weight loss of 14.1%, which represents further degradation of any remaining polymer structures until the process reaches completion.

For PVC-PVA@CS QD membrane, the initial weight loss is around 16.3% (first decomposition stage), slightly lower than the PVC-PVA membrane. This suggests that the membrane is losing moisture and volatile components, but the presence of chitosan quantum dots might be stabilizing the structure to some extent. At second decomposition stage, the major weight loss occurs at 52.02%, indicating the primary degradation of the polymer matrix. The reduced weight loss in this stage compared to the PVC-PVA membrane suggests that the chitosan quantum dots contribute to thermal stabilization, delaying complete degradation. The final weight loss of 19.86% is observed (final stage) as the remaining polymer materials degrade. Notably, the residue left after complete decomposition is 6.69%, indicating that a portion of the membrane remains thermally stable due to the quantum dots. The PVC-PVA@CS QD membrane demonstrates improved thermal stability compared to the PVC-PVA membrane. This is evident from the lower weight loss percentages during the major decomposition stages. The presence of chitosan quantum dots appears to reinforce the membrane structure, making it more resistant to thermal degradation.

### 3.7. Electrical properties

Understanding the electrical properties of nanofiltration (NF) membranes is essential for elucidating their ion transport behavior, interfacial characteristics, and functional performance in desalination applications. Although NF membranes primarily rely on size exclusion and charge-based separation mechanisms, their efficiency is also significantly influenced by ionic mobility and electrostatic interactions within the membrane matrix. Measuring electrical conductivity, charge transfer resistance, and dielectric properties provides insight into the membrane's ability to facilitate ion migration, store electrical energy, and resist fouling. These properties are particularly critical in hybrid membranes incorporating conductive or polar nanomaterials such as chitosan quantum dots (CS QDs), which can modify the membrane's charge distribution and enhance its interaction with ionic species. Therefore, a comprehensive electrical characterization of the fabricated PVC-PVA@CS QD membrane was conducted to assess its potential for advanced water purification applications.^52–54^

#### 3.7.1 Electrical properties

The electrical properties of the PVA-PVC@CS QD membrane were evaluated by measuring electrical conductivity and ion transport characteristics through electrochemical impedance spectroscopy (EIS). The results are presented in Table 3. The electrical conductivity of the PVA-PVC@CS QD membrane was found to be 1.2 × 10^−3^ S cm^−1^, a noticeable improvement over the 8.5 × 10^−4^ S cm^−1^ for the neat PVC-PVA membrane. This enhancement is likely due to the increased ionic pathways provided by the chitosan QDs, which facilitate easier movement of ions through the membrane. Additionally, EIS measurements indicated a noticeable decrease in charge transfer resistance for the PVA-PVC@CS QD membrane. The charge transfer resistance was decreased from 150 Ω in the neat PVC-PVA membrane to 95 Ω in the PVA-PVC@CS QD membranes, reflecting enhanced ion mobility.^55,56^

**Table 3:** Electrical properties of PVC-PVA and PVA-PVC@CS QD membrane

Property	Neat PVC-PVA membrane	PVA-PVC@CS QD membrane
Electrical conductivity	8.5 × 10^−4^ S cm^−1^	1.2 × 10^−3^ S cm^−1^
Charge transfer resistance	150 Ω	95 Ω

These results suggest that the presence of chitosan QDs not only improves conductivity but also enhances the overall efficiency of ion transport within the membrane. Where, improved electrical conductivity and reduced charge transfer resistance are vital for the efficiency of desalination processes, particularly in electrochemical applications such as capacitive deionization. The enhanced ion transport properties ensure that the membrane can effectively separate ions from water, improving the overall desalination performance. This leads to more efficient water purification, with higher throughput and lower energy consumption. The designed PVA-PVC@CS QD membrane offer a promising solution for sustainable and efficient desalination, contributing to the development of more durable and high-performance water treatment technologies.^57,58^

#### 3.7.2 Dielectric properties

**Fig. 12:**
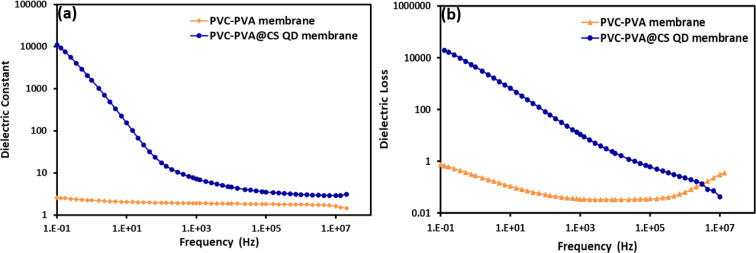
(a and b): (a) The dielectric constant, and (b) dielectric loss as a function of frequency of PVC-PVA and PVC-PVA@CS QD membranes.

Fig. 12a and b show the dielectric constant and dielectric loss, as functions of frequency for both PVC-PVA and PVC-PVA@CS QD membranes. The dielectric constant of the PVC-PVA membrane remains relatively low and stable across the frequency range, showing minimal variation. This indicates that the PVC-PVA membrane has a relatively low ability to store electrical energy within its structure, which is typical for polymeric materials with limited polarizability. The dielectric constant for the PVC-PVA@CS QD membrane is significantly higher, particularly at lower frequencies, and decreases as the frequency increases. This behavior is characteristic of materials with high polarizability, where the presence of chitosan quantum dots enhances the overall dielectric response. The decrease in dielectric constant with frequency is owing to the inability of dipoles to align with the rapidly changing electric field at higher frequencies.^59,60^

Dielectric loss of the PVC-PVA membrane remains low across the frequency spectrum, indicating minimal energy dissipation as heat. This suggests that the PVC-PVA membrane exhibits stable insulating properties with low electrical conductivity. The dielectric loss for the PVC-PVA@CS QD membrane is higher than that of the PVC-PVA membrane, particularly at lower frequencies. This higher dielectric loss suggests increased energy dissipation, likely due to the enhanced polarization effects caused by the chitosan quantum dots. As the frequency increases, the dielectric loss decreases, indicating that at higher frequencies, the material's ability to dissipate energy diminishes.^60^

Overall, the incorporation of chitosan quantum dots significantly enhances the dielectric properties of the PVC-PVA membrane, as seen in the higher dielectric constant and dielectric loss values. This makes the PVC-PVA@CS QD membrane more suitable for applications where higher dielectric performance is required, such as in sensors or capacitors. Conversely, the PVC-PVA membrane, with its stable dielectric properties, may be better suited for applications requiring consistent insulating behavior.

## 4. Conclusion

In conclusion, the study highlights the significant advancements achieved by incorporating chitosan quantum dots (CS QDs) into the PVC-PVA membrane matrix, resulting in a multifunctional membrane with enhanced mechanical, thermal, electrical, and scale inhibition properties. The PVC-PVA@CS QD membrane demonstrated superior tensile strength, elongation at break, and thermal stability, indicating its ability to withstand the rigorous conditions of desalination processes. The presence of CS QDs not only reinforced the membrane structure but also improved its thermal degradation resistance, making it a robust candidate for long-term applications in water purification.

Moreover, the electrical properties of the PVC-PVA@CS QD membrane were markedly improved, as evidenced by higher electrical conductivity and reduced charge transfer resistance. The dielectric analysis further underscored the enhanced polarization and energy dissipation capabilities of the membrane, attributed to the synergistic interactions between the polymer matrix and the CS QDs. The membrane's superior scale inhibition performance, particularly against calcite formation, confirms its potential for efficient and sustainable desalination processes. Overall, the PVC-PVA@CS QD membrane represents a significant advancement in membrane technology, offering a comprehensive solution to the challenges of water purification and scale formation inhibition in desalination applications.

## Data availability

Data will be available upon request.

## Conflicts of interest

The authors declare that they have no known competing financial interests or personal relationships that could have appeared to influence the work reported in this paper.
